# Circulating JKAP levels may correlate with postpartum anxiety and depression through its interaction with T helper 17 cells

**DOI:** 10.1590/1414-431X2024e13253

**Published:** 2024-10-07

**Authors:** Jing Zhang, Cui Li, Zhuo Chen, Xiaoling Zhao, Yuanyuan He, Lei Wei, Nana Kong

**Affiliations:** 1 Department of Obstetrics, Xingtai People's Hospital, Xingtai, China; 2 Department of Operating Room, Xingtai People's Hospital, Xingtai, China; 3 Department of Cancer Laboratory, Xingtai People's Hospital, Xingtai, China; 4 Department of Pediatrics, Xingtai People's Hospital, Xingtai, China; 5 Department of Cardiovascular Surgery, Shanxi Provincial People's Hospital, Taiyuan, China

**Keywords:** Postpartum women, Jun N-terminal kinase pathway-associated phosphatase, T helper 17 cells, Anxiety, Depression

## Abstract

Jun N-terminal kinase pathway-associated phosphatase (JKAP) regulates CD4^+^ T-cell differentiation and immunity, which are linked to mental disorders. This study aimed to explore the relationships between JKAP and T helper 17 (Th17)/regulatory T (Treg) ratio, as well as their associations with anxiety and depression in postpartum women. Serum JKAP were measured by enzyme-linked immunosorbent assay and blood Th17 and Treg cells were measured by flow cytometry in 250 postpartum women. Anxiety and depression were evaluated by the 6-item State-Trait Anxiety Inventory (STAI6) and Edinburgh Postnatal Depression Scale (EPDS). Anxiety and depression rates were 22.0 and 28.4%, respectively, among postpartum women. Notably, JKAP was negatively associated with the STAI6 (P=0.002) and EPDS scores (P<0.001) in postpartum women and was lower in postpartum women with anxiety (P=0.023) or depression (P=0.002) than in those without. Moreover, JKAP was inversely related to Th17 cells and Th17/Treg ratio but positively correlated with Treg cells in postpartum women (all P<0.001). Interestingly, Th17 cells and Th17/Treg ratio were both positively associated with STAI6 and EPDS scores in postpartum women (all P<0.001). Furthermore, Th17 cells and Th17/Treg ratio were lower in postpartum women with anxiety or depression than in those without (all P<0.01). Nevertheless, Treg cells were not linked to anxiety or depression in postpartum women. JKAP was negatively associated with Th17 cells and Th17/Treg ratio; moreover, they all related to anxiety and depression in postpartum women, indicating that JKAP may be involved in postpartum anxiety and depression via interactions with Th17 cells.

## Introduction

Anxiety and depression are prevalent psychological issues in postpartum women (1,2). These psychological problems negatively affect postpartum women's mental state and physical rehabilitation, even resulting in self‐harm and suicide (3-5). The occurrence of anxiety and depression in postpartum women is associated with many factors, which mainly include postpartum complications, hormonal imbalances, and immune-inflammatory abnormalities (6-9). Notably, current evidence suggests that T cells may play an important role in immune-inflammatory processes and are involved in the development of anxiety and depression (10,11). Specifically, human T helper 17 (Th17) cells secrete the proinflammatory cytokines interleukin (IL)-17 and IL-22, which impair the blood-brain barrier and kill human neurons *in vivo* and *in vitro*, thereby recruiting cluster of differentiation 4^+^ (CD4^+^) lymphocytes and promoting central nervous system inflammation (12). Regulatory T (Treg) cells suppress inflammatory responses and maintain immune homeostasis, and an imbalance in the Th17/Treg ratio causes immune dysregulation and increases inflammation (13). Immune-inflammatory abnormalities induce serotonin dysregulation, hypothalamus-pituitary-adrenal axis dysregulation, and white and gray matter pathology, which are involved in the occurrence of anxiety and depression (14). Moreover, some previous studies have revealed that immune-inflammatory processes are related to anxiety and depression in postpartum women (8,15). Therefore, exploring potential immune-inflammatory mechanisms involved in the occurrence of anxiety and depression, especially the regulation of Th17 and Treg cells, might contribute to the management of postpartum women.

Jun N-terminal kinase (JNK) pathway-associated phosphatase (JKAP) is involved in regulating the immune system by inhibiting the differentiation of Th17 cells (16-18). Notably, the interconnection of the JKAP with Th17 cells in patients with several neurological diseases has been explored in previous studies (19-21). For example, one recent study illustrated that the JKAP is inversely associated with Th17 cells in Alzheimer's disease patients (20). Moreover, another study also revealed a negative correlation between JKAP and Th17 cells in patients with Parkinson's disease (19). More importantly, one study not only showed that the reduction in JKAP is related to elevated interleukin (IL)‐17A but also revealed that both are linked to anxiety risk in acute ischemic stroke patients (21). On the basis of the above research, a reasonable assumption is that JKAP and Th17 cells may be correlated and associated with mental health in postpartum women. However, relevant research is still lacking.

Therefore, our study aimed to explore the relationships between JKAP and Th17 cells, as well as their associations with anxiety and depression in postpartum women.

## Material and Methods

### Participants

A total of 250 postpartum women who delivered at Xingtai People's Hospital (China) between March 2022 and December 2022 were enrolled in this study. The inclusion criteria were as follows: i) aged more than 18 years; ii) had a normal pregnancy and delivery; iii) had the ability and willingness to complete the 6-item State-Trait Anxiety Inventory (STAI6) (22) and Edinburgh Postnatal Depression Scale (EPDS) (23); and iv) were willing to provide a blood sample for scientific research. The exclusion criteria were as follows: i) had complications within 6 weeks after delivery; ii) had malignant diseases; iii) had mental disorders, e.g. schizophrenia, neuroticism, or bipolar disorder, making them unable to cooperate with this study; and iv) had inflammatory or autoimmune diseases. There was no restriction on the medication of participants. This study received the approval of the Ethics Committee of the Xingtai People's Hospital. All participants provided informed consent.

### Data collection

The following clinical characteristics of postpartum women were collected: age, education level, annual family income, history of smoking, history of drinking, previous gravidity, previous birth number, number of previous abortions, gestational week, unplanned pregnancy, planned maternity leave time, satisfaction with labor experience, breastfeeding difficulty, and number of caregivers.

### Sample collection and detection

Peripheral blood (PB) samples were collected from postpartum women at 6 weeks after delivery. The collected PB samples were divided into two parts: one was used for analyzing Th17 and Treg cells quantitatively and the other was used for isolating serum to detect JKAP.

For Th17 and Treg cell quantification, CD4^+^ T cells were separated from the PB samples via a Dynabeads™ FlowComp™ Human CD4 kit (No. Cat. 11361D, Thermo Fisher Scientific, USA). A total of 5-7×10^5^ cells/mL were obtained. Th17 (CD4+IL-17A+) and Treg (CD25+FoxP3+) cell quantification was performed by flow cytometry (FCM). Commercial kits were purchased from R&D Systems (USA; catalog numbers FMC007B and FMC021). Then, the Th17/Treg ratio was calculated. An LSRFortessa flow cytometer (BD, USA) was used to analyze the cells, and FlowJo X (BD) was used to analyze the data. The gating strategy for Th17 and Treg cells was as follows: for Th17 cells, CD4+IL-17A+ dots were gated; for Treg cells, CD4+CD25+FoxP3+ dots were sorted.

For JKAP detection, an enzyme-linked immunosorbent assay (ELISA) was performed, and commercial kits (No. MBS9915719, MyBioSource, USA) were used. The reference technique of the kit was strictly followed for all experiments.

### Evaluation

Postpartum women completed the STAI6 and EPDS questionnaires at 6 weeks after delivery. The STAI6 questionnaire contained 6 items and the total score ranged from 6 to 24 (1 to 4 scores per item), and the EPDS questionnaire contained 10 items and the total score ranged from 0 to 30 (0 to 3 scores per item). Based on the results of the questionnaires, postpartum anxiety and depression were evaluated. Postpartum anxiety was defined as a total STAI6 score greater than 15, and postpartum depression was defined as a total EPDS score greater than 12 (8).

### Statistics

SPSS v.26.0 (IBM, USA) and GraphPad Prism v.7.0 (GraphPad Prism, USA) were used for analysis and plotting. The Wilcoxon rank-sum test or Kruskal-Wallis H rank-sum test was used for comparison analysis as appropriate; the Spearman test was used for correlation analysis. P<0.05 was considered to indicate statistical significance.

## Results

### Characteristics of postpartum women

The mean age of the postpartum women was 28.2±3.7 years. Moreover, the median [interquartile range (IQR)] number of gestational weeks was 39.0 (38.0-40.0) weeks. Notably, the medians (IQRs) of Th17 cells, Treg cells, and the Th17/Treg ratio were 2.4 (1.4-3.9)%, 6.5 (5.3-7.6)%, and 0.4 (0.2-0.6), respectively. More detailed clinical information is listed in Table 1.

**Table 1:** Clinical characteristics of postpartum women.

Characteristics	Values (n=250)
Age (years), mean±SD	28.2±3.7
Education level, n (%)	
Primary school or less	9 (3.6)
Middle or high school	149 (59.6)
Undergraduate or above	92 (36.8)
Annual family income (CYN), n (%)	
<100,000	9 (3.6)
100,000-199,999	141 (56.4)
200,000-299,999	71 (28.4)
≥300.000	29 (11.6)
History of smoking, n (%)	
No	215 (86.0)
Yes	35 (14.0)
History of drinking, n (%)	
No	195 (78.0)
Yes	55 (22.0)
Previous gravidity times, n (%)	
0	48 (19.2)
1	102 (40.8)
2	77 (30.8)
3	20 (8.0)
4	3 (1.2)
Previous birth number, n (%)	
0	177 (70.8)
1	71 (28.4)
2	2 (0.8)
Previous abortion times, n (%)	
0	63 (25.2)
1	126 (50.4)
2	56 (22.4)
3	5 (2.0)
Gestational week, median (IQR)	39.0 (38.0-40.0)
Unplanned pregnancy, n (%)	
No	218 (87.2)
Yes	32 (12.8)
Planned maternity leave time, n (%)	
≤128 days	179 (71.6)
>128 days	71 (28.4)
Satisfaction with labor experience, n (%)	
No	60 (24.0)
Yes	190 (76.0)
Breastfeeding difficulty, n (%)	
No	158 (63.2)
Yes	92 (36.8)
Number of caregivers, n (%)	
<2	77 (30.8)
≥2	173 (69.2)
Th17 cell (%), median (IQR)	2.4 (1.4-3.9)
Treg cell (%), median (IQR)	6.5 (5.3-7.6)
Th17/Treg ratio, median (IQR)	0.4 (0.2-0.6)

SD: standard deviation; CYN: Chinese Yuan; IQR: interquartile range; Th17: T helper type 17; Treg: regulatory T.

### JKAP distribution and its relationship with clinical characteristics

**Figure 1:**
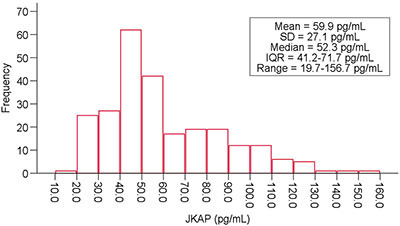
The distribution of the JKAP in 250 postpartum women.

The distribution of JKAP in postpartum women was right-skewed. Moreover, the median (IQR) concentration of JKAP was 52.3 (41.2-71.7) pg/mL, ranging from 19.7 to 156.7 pg/mL (Figure 1). Interestingly, increased JKAP was associated with higher education levels (P=0.026). However, there was no correlation between JKAP and other clinical characteristics, such as age, annual family income, or gestational week (all P>0.05) (Table 2).

**Table 2:** Correlation of JKAP with clinical characteristics in postpartum women.

Characteristics	JKAP (pg/mL), median (IQR)	P value
Age		0.247
<28 years	55.0 (42.6-83.5)	
≥28 years	50.2 (40.8-74.0)	
Education level		0.026
Primary school or less	40.2 (37.6-49.8)	
Middle or high school	50.3 (39.4-74.7)	
Undergraduate or above	55.1 (44.0-86.1)	
Annual family income (CYN)		0.183
<100,000	41.9 (35.1-74.0)	
100,000-199,999	49.8 (37.9-75.2)	
200,000-299,999	53.8 (43.6-78.3)	
≥300,000	59.5 (45.0-83.9)	
History of smoking		0.349
No	51.4 (40.5-74.9)	
Yes	55.5 (43.7-84.1)	
History of drinking		0.093
No	52.8 (42.0-80.7)	
Yes	47.8 (38.9-72.4)	
Previous gravidity times		0.898
0	52.2 (40.7-75.8)	
1	53.8 (38.4-83.5)	
2	50.2 (42.5-77.1)	
3	54.7 (45.5-69.7)	
4	38.1 (31.7-NA)	
Previous birth number		0.253
0	53.7 (40.9-82.6)	
1	49.3 (41.1-61.4)	
2	88.8 (59.7-NA)	
Previous abortion times		0.367
0	51.2 (39.2-76.1)	
1	52.4 (41.6-79.1)	
2	56.8 (43.8-81.5)	
3	45.0 (34.9-51.8)	
Gestational week		0.689
<39.0 weeks	52.7 (40.7-76.4)	
≥39.0 weeks	52.1 (41.7-77.3)	
Unplanned pregnancy		0.377
No	52.9 (41.2-77.6)	
Yes	48.1 (39.9-72.7)	
Planned maternity leave time		0.424
≤128 days	50.8 (41.2-74.5)	
>128 days	53.8 (40.2-84.2)	
Satisfaction with labor experience		0.859
No	52.2 (42.3-75.4)	
Yes	52.3 (40.4-78.5)	
Breastfeeding difficulty		0.624
No	52.9 (41.6-78.4)	
Yes	50.1 (41.0-76.0)	
Number of caregivers		0.918
<2	52.4 (43.2-71.1)	
≥2	51.4 (40.5-82.6)	

JKAP: Jun N-terminal kinase pathway-associated phosphatase; IQR: interquartile range; CYN: Chinese Yuan; NA: not available. Age and gestational week were dichotomized by median values. The median value of age was 28.0 years and the median value of gestational week was 39.0 weeks. Wilcoxon rank-sum test, Kruskal-Wallis H rank-sum test, or Spearman test.

### Association of JKAP with anxiety and depression

**Figure 2:**
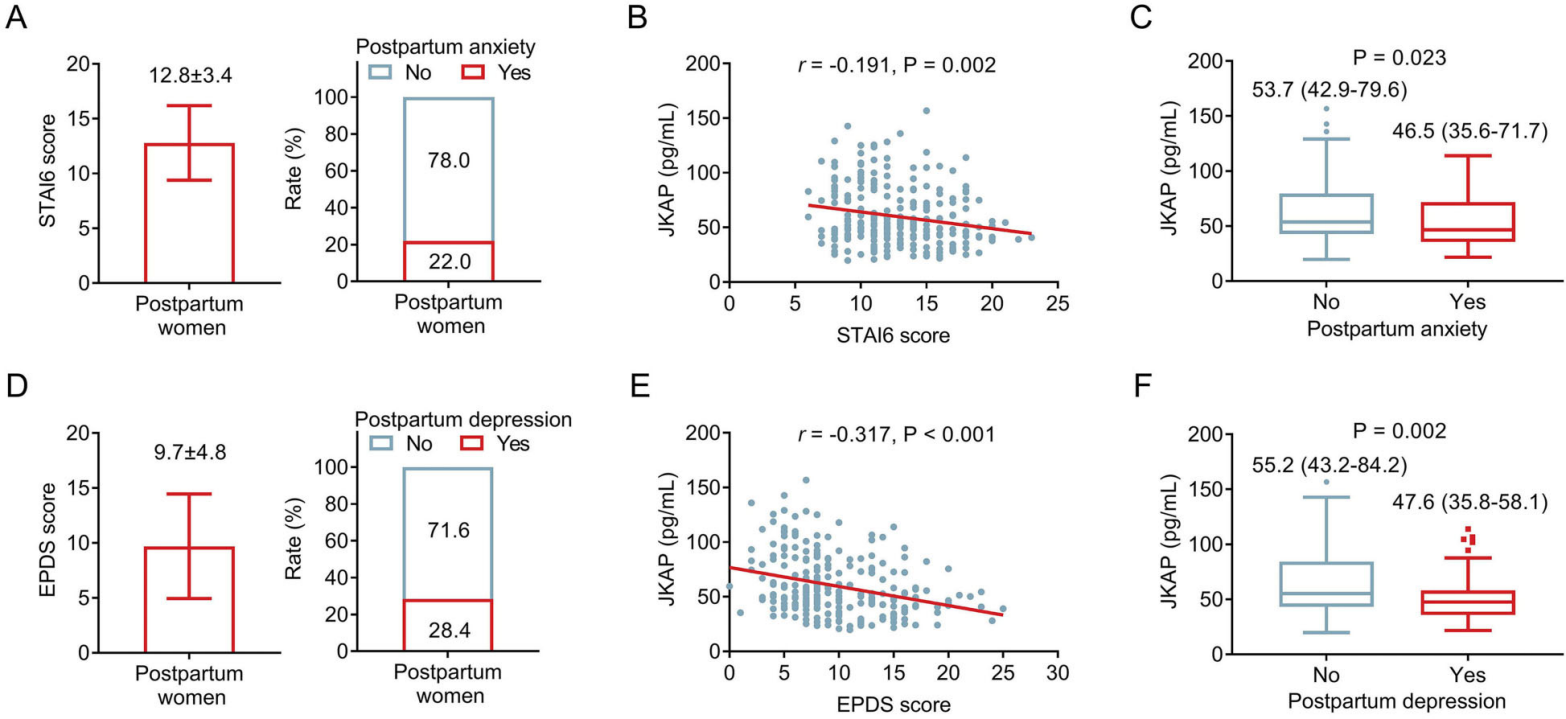
JKAP in postpartum women with and without anxiety or depression. **A**, State-Trait Anxiety Inventory (STAI6) score and anxiety rate. Associations of JKAP with STAI6 score (**B**) and JKAP with anxiety classified by STAI6 score (**C**); associations of the EPDS score and depression rate (**D**); and the associations of JKAP with the EPDS score (**E**) and depression classified by the EPDS score (**F**) in postpartum women. Data are reported as means (SD) or median (interquartile range). Wilcoxon rank-sum test.

The mean STAI6 score in postpartum women was 12.8±3.4. Based on the cutoff STAI6 score of 15, 22.0% of the women had anxiety, and 78.0% had no anxiety (Figure 2A). Moreover, JKAP was negatively related to the STAI6 score (r=-0.191, P=0.002) (Figure 2B). Notably, JKAP was lower in postpartum women with anxiety than in those without anxiety [median (IQR): 46.5 (35.6-71.7) *vs* 53.7 (42.9-79.6) pg/mL] (P=0.023) (Figure 2C).

The mean EPDS score in postpartum women was 9.7±4.8. According to the cutoff EPDS score of 12, 28.4% had depression and 71.6% had no depression (Figure 2D). Furthermore, JKAP was inversely linked with the EPDS score (r=-0.317, P<0.001) (Figure 2E). In addition, JKAP was lower in postpartum women with depression than in those without depression [median (IQR): 47.6 (35.8-58.1) *vs* 55.2 (43.2-84.2) pg/mL] (P=0.002) (Figure 2F).

### Association of JKAP with Th17 cells, Treg cells, and the Th17/Treg ratio

**Figure 3:**
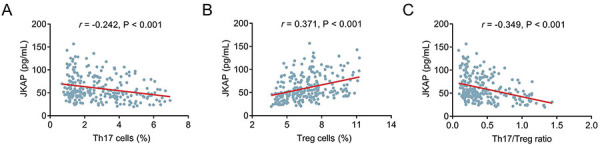
**A-C**, Spearman correlation analysis of JKAP with Th17 cells, Treg cells, and the Th17/Treg ratio in 250 postpartum women.

Interestingly, the correlation analysis revealed that JKAP was negatively correlated with Th17 cells in postpartum women (r=-0.242, P<0.001) (Figure 3A). However, JKAP was positively correlated to Treg cells (r=0.371, P<0.001) (Figure 3B). In addition, JKAP was inversely correlated with the Th17/Treg ratio (r=-0.349, P<0.001) (Figure 3C).

### Association of Th17 cells, Treg cells, and the Th17/Treg ratio with anxiety

**Figure 4:**
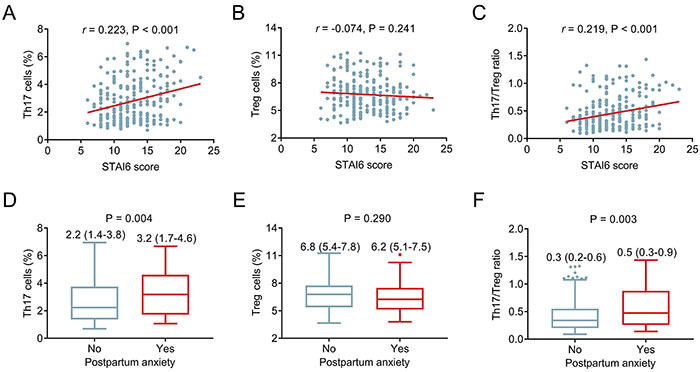
Th17 cells, Treg cells, and the Th17/Treg ratio in postpartum women with and without anxiety. **A**-**C**, Spearman correlation of Th17 cells, Treg cells, and the Th17/Treg ratio with the State-Trait Anxiety Inventory (STAI6) score, and **D**-**F**, their levels in women with and without anxiety classified by the STAI6 score. Data are reported as median (interquartile range). Wilcoxon rank-sum test.

Th17 cells were positively correlated with STAI6 score (r=0.223, P<0.001) (Figure 4A), while Treg cells were not correlated to STAI6 score in postpartum women (r=-0.074, P=0.241) (Figure 4B). Moreover, the Th17/Treg ratio was positively correlated with the STAI6 score (r=0.219, P<0.001) (Figure 4C). Th17 cell levels were greater in postpartum women with anxiety than in those without anxiety [median (IQR): 3.2 (1.7-4.6) *vs* 2.2 (1.4-3.8)%] (P=0.004) (Figure 4D). Nevertheless, there was no difference in Treg cells between postpartum women with and without anxiety [median (IQR): 6.2 (5.1-7.5) *vs* 6.8 (5.4-7.8)%] (P=0.290) (Figure 4E). Notably, the Th17/Treg ratio was greater in postpartum women with anxiety than in those without anxiety [median (IQR): 0.5 (0.3-0.9) *vs* 0.3 (0.2-0.6)] (P=0.003) (Figure 4F).

### Association of Th17 cells, Treg cells, and the Th17/Treg ratio with depression

**Figure 5:**
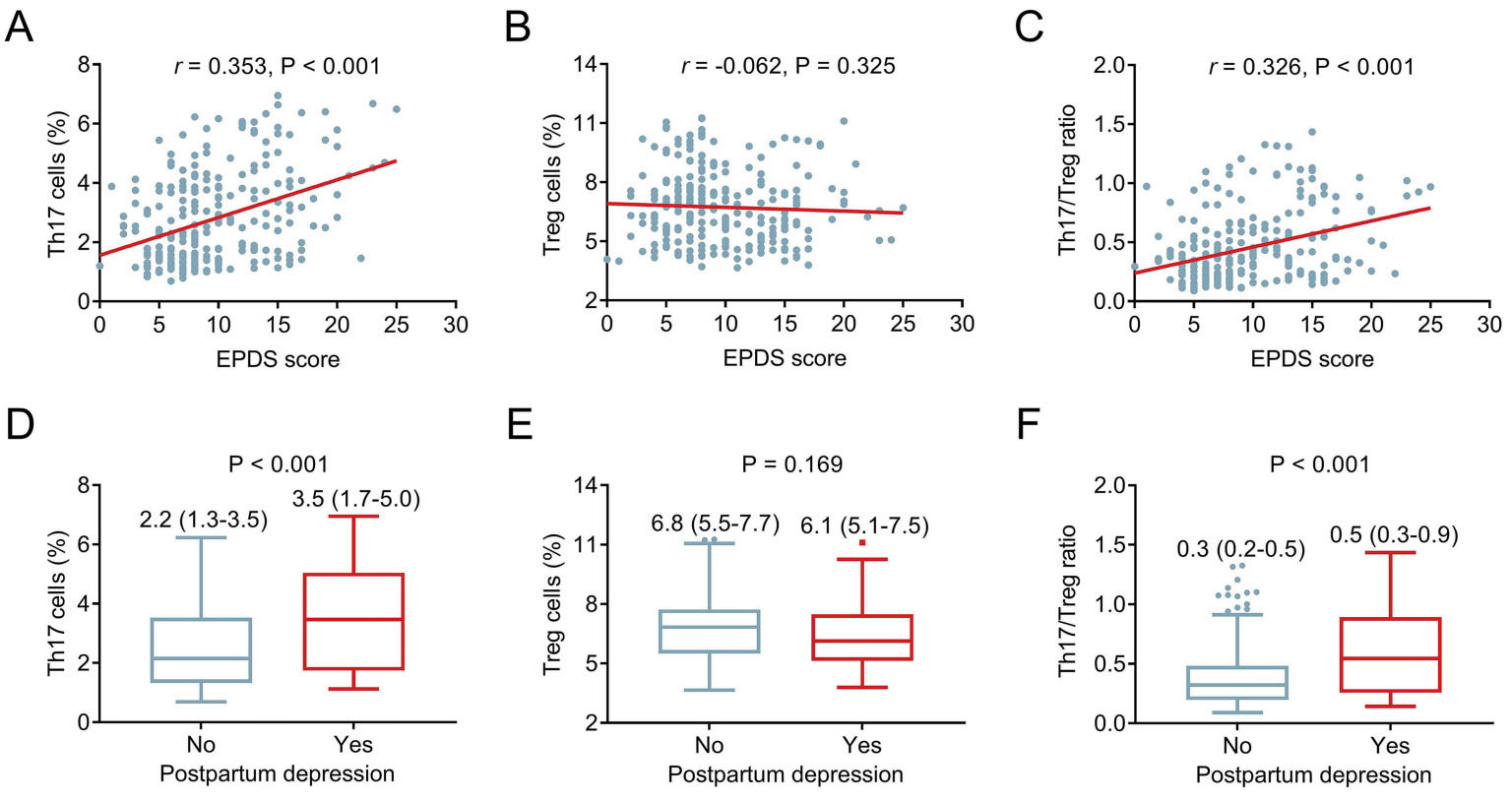
Th17 cells, Treg cells, and the Th17/Treg ratio in postpartum women with and without depression. **A**-**C**, Spearman correlation of Edinburgh Postnatal Depression Scale (EPDS) with Th17 cells, Treg cells, and the Th17/Treg ratio and, **D**-**F**, their levels in women with and without depression classified by the EPDS score. Data are reported as median (interquartile range). Wilcoxon rank-sum test.

A positive correlation was observed between Th17 cells and the EPDS score in postpartum women (r=0.353, P<0.001) (Figure 5A). However, no relationship was found between Treg cells and the EPDS score in postpartum women (r=-0.062, P=0.325) (Figure 5B). Furthermore, the Th17/Treg ratio was positively associated with the EPDS score (r=0.326, P<0.001) (Figure 5C). Th17 cells were greater in women with depression than in those without depression [median (IQR): 3.5 (1.7-5.0) *vs* 2.2 (1.3-3.5)%] (P<0.001) (Figure 5D). No difference in Treg cells was detected between participants with and without depression [median (IQR): 6.1 (5.1-7.5) *vs* 6.8 (5.5-7.7)%] (P=0.169) (Figure 5E). Moreover, the Th17/Treg ratio was greater in participants with depression than in those without depression [median (IQR): 0.5 (0.3-0.9) *vs* 0.3 (0.2-0.5)] (P<0.001) (Figure 5F).

## Discussion

Anxiety and depression have already become non-negligible psychological issues in postpartum women. Previous studies have suggested that the rates of anxiety and depression in postpartum women are approximately 20.6-27.9% and 20.4-29.1%, respectively (8,24-26). The findings of our study were similar showing that 22.0 and 28.4% of postpartum women experienced anxiety and depression, respectively. Notably, one previous study showed even greater anxiety and depression rates in postpartum women, reaching 40.8 and 38.8%, respectively (27). Another study showed that anxiety and depression rates in postpartum women were lower, at only 14.7 and 8.0%, respectively (2). These different findings might be due to the following reasons. 1) The use of different time points for evaluating postpartum women's anxiety and depression; 2) The differences in the included population; 3) Our study excluded postpartum women who experienced complications within 6 weeks after delivery. Therefore, a comprehensive evaluation that considers these factors should be conducted to obtain more accurate results.

JKAP may be involved in the regulation of anxiety and depression (28). However, the relationship of JAKP with anxiety and depression in postpartum women is still unclear. Our study showed that there was a negative relationship between the JKAP and anxiety and depression in postpartum women. According to several previous studies, JKAP suppressed the activation of the IL-6/signal transducer and activator of transcription 3 (STAT3) pathway in mice (29); moreover, inhibition of the IL-6/STAT3 pathway prevented acid phosphatase 5 upregulation, which modulated the excitability of prelimbic cortex pyramidal neurons and reduced depression in rats (30). Furthermore, JKAP reduced the secretion of IL-17A by inhibiting the differentiation of human Th17 cells (17); moreover, the reduction in IL-17A suppressed excitotoxic hippocampal neuronal death and reduced the production of hilar ectopic granule neurons in mice, which might alleviate anxiety and depression (31). These studies suggest that JKAP can negatively regulate anxiety and depression (17,29-31).

Notably, JKAP inhibits immune responses in various diseases (16,17,32). For example, one previous study revealed that JKAP restrains the differentiation of Th17 cells in inflammatory bowel disease (17). Another study revealed that the knockout of JKAP can mediate T-cell hyperactivation in mice (32). Moreover, the association of JKAP with Th17 cells has also been reported in some clinical studies (17,33,34). Our study revealed that JKAP was negatively associated with Th17 cells and the Th17/Treg ratio, but positively associated to Treg cells in postpartum women. Several previous studies support the findings of our study: JKAP inactivates lymphocyte-specific protein tyrosine kinase and blocks the transmission of T-cell receptor (TCR) signaling, which further inhibits the differentiation of CD4^+^ T cells into Th17 cells in mice (16). Moreover, attenuation of TCR signaling promoted the differentiation of Treg cells in mice (35). Therefore, the association of JKAP with Th17 and Treg cells in postpartum women might be explained by the effect of JKAP on TCR signaling regulation. Because JKAP can facilitate the differentiation of Th17 cells and inhibit the differentiation of Treg cells, there was a negative correlation between JKAP and the Th17/Treg ratio in postpartum women.

Previous evidence has shown that Treg cells are involved in systemic inflammation and neuroimmune dysfunction and illustrated the possibility of Treg cells guiding personalized treatment of mental disorders in the future (36,37). In addition, one previous study revealed that inflammation is linked to anxiety in postpartum women (15). Another study illustrated that blood Th17 cells and IL-17A levels are positively related to anxiety and depression in postpartum women (8). The findings of our study were similar to those of these previous studies, which revealed that Th17 cells and the Th17/Treg ratio were positively associated with anxiety and depression in postpartum women. One possible explanation is that human Th17 cells secrete IL-17 and IL-22, which destroy the integrity of the blood-brain barrier and promote inflammation, thereby leading to anxiety and depression (12).

Although one previous study revealed a negative correlation between JKAP and anxiety and depression, the causal relationships between JKAP and anxiety and depression are still unclear (21). Interestingly, a novel finding in our study was that there were differences in the levels of JKAP among postpartum women with different levels of education. We speculated that this result might be due to differences in education levels leading to differences in anxiety and depression in postpartum women (38); moreover, anxiety and depression might affect JKAP levels. Therefore, elevated JKAP was correlated with higher education levels in postpartum women. However, these hypotheses need to be further explored in future research.

There were a few limitations in the current study. 1) The STAI6 score and EPDS score were self-reported by the enrolled postpartum women, which might have led to a certain degree of bias; 2) The specific mechanism by which JKAP participates in anxiety and depression needs to be further explored in future research; 3) One previous study showed that the detection of biomarkers in cerebrospinal fluid is more meaningful for predicting, identifying, and treating depression early in postpartum women than in plasma (39). Our study only obtained serum JKAP, and JKAP from cerebrospinal fluid samples from postpartum women should be detected in future research for further investigation; 4) In our study, only Th17 cells and Treg cells were detected at 6 weeks after delivery, and future research is required to measure Th17 cells and Treg cells in postpartum women at multiple time points after delivery.

In conclusion, JKAP was inversely associated to Th17 cells and Th17/Treg ratio, and all of them are associated with anxiety and depression in postpartum women. Our study showed that JKAP may be involved in the development of postpartum anxiety and depression by interacting with Th17 cells and the use of JKAP and Th17 cells might be beneficial for the management of postpartum women.

## References

[1] Fleischman EK, Connelly CD, Calero P (2022). Depression and anxiety, stigma, and social support among women in the postpartum period. Nurs Womens Health.

[2] van der Zee-van den Berg AI, Boere-Boonekamp MM, Groothuis-Oudshoorn CGM, Reijneveld SA (2021). Postpartum depression and anxiety: a community-based study on risk factors before, during and after pregnancy. J Affect Disord.

[3] Liu C, Xiao D, Han D, Li S, Zhu T, Wang W (2021). Effects of cognitive nursing combined with continuous nursing on postpartum mental state and rehabilitation. Biomed Res Int.

[B04] Chin K, Wendt A, Bennett IM, Bhat A (2022). Suicide and maternal mortality. Curr Psychiatry Rep.

[5] Lee YL, Tien Y, Bai YS, Lin CK, Yin CS, Chung CH (2022). Association of postpartum depression with maternal suicide: a nationwide population-based study. Int J Environ Res Public Health.

[6] Koutra K, Vassilaki M, Georgiou V, Koutis A, Bitsios P, Kogevinas M (2018). Pregnancy, perinatal and postpartum complications as determinants of postpartum depression: the Rhea mother-child cohort in Crete, Greece. Epidemiol Psychiatr Sci.

[B07] Hedges VL, Heaton EC, Amaral C, Benedetto LE, Bodie CL, D'Antonio BI (2021). Estrogen withdrawal increases postpartum anxiety via oxytocin plasticity in the paraventricular hypothalamus and dorsal raphe nucleus. Biol Psychiatry.

[B08] Min Z, Li Y, Ying H (2022). Blood T-helper 17 cells and interleukin-17A correlate with the elevated risk of postpartum depression and anxiety. J Clin Lab Anal.

[9] Bränn E, Fransson E, White RA, Papadopoulos FC, Edvinsson A, Kamali-Moghaddam M (2020). Inflammatory markers in women with postpartum depressive symptoms. J Neurosci Res.

[10] Miller AH (2010). Depression and immunity: a role for T cells?. Brain Behav Immun.

[11] Osborne LM, Brar A, Klein SL (2019). The role of Th17 cells in the pathophysiology of pregnancy and perinatal mood and anxiety disorders. Brain Behav Immun.

[12] Kebir H, Kreymborg K, Ifergan I, Dodelet-Devillers A, Cayrol R, Bernard M (2007). Human TH17 lymphocytes promote blood-brain barrier disruption and central nervous system inflammation. Nat Med.

[13] Lee GR (2018). The balance of Th17 *versus* Treg cells in autoimmunity. Int J Mol Sci.

[14] McNamara RK, Lotrich FE (2012). Elevated immune-inflammatory signaling in mood disorders: a new therapeutic target?. Expert Rev Neurother.

[15] Groer M, Louis-Jacques A, Szalacha L, Redwine L, Dracxler R, Keefe D (2020). Relationship of anxiety, inflammation, and telomere length in postpartum women: a pilot study. Biol Res Nurs.

[16] Li JP, Yang CY, Chuang HC, Lan JL, Chen DY, Chen YM (2014). The phosphatase JKAP/DUSP22 inhibits T-cell receptor signalling and autoimmunity by inactivating Lck. Nat Commun.

[B17] Zhou R, Chang Y, Liu J, Chen M, Wang H, Huang M (2017). JNK pathway-associated phosphatase/DUSP22 suppresses CD4(+) T-Cell activation and Th1/Th17-Cell differentiation and negatively correlates with clinical activity in inflammatory bowel disease. Front Immunol.

[18] Chuang HC, Tan TH (2019). MAP4K Family kinases and DUSP family phosphatases in T-Cell signaling and systemic lupus erythematosus. Cells.

[19] Yang Q, Zhuang J, Cai P, Li L, Wang R, Chen Z (2021). JKAP relates to disease risk, severity, and Th1 and Th17 differentiation in Parkinson's disease. Ann Clin Transl Neurol.

[B20] Zeng J, Liu J, Qu Q, Zhao X, Zhang J (2022). JKAP, Th1 cells, and Th17 cells are dysregulated and inter-correlated, among them JKAP and Th17 cells relate to cognitive impairment progression in Alzheimer's disease patients. Ir J Med Sci.

[21] Wang C, Huo H, Li J, Zhang W, Liu C, Jin B (2022). The longitudinal changes of serum JKAP and IL-17A, and their linkage with anxiety, depression, and cognitive impairment in acute ischemic stroke patients. J Clin Lab Anal.

[22] Marteau TM, Bekker H (1992). The development of a six-item short-form of the state scale of the Spielberger State-Trait Anxiety Inventory (STAI). Br J Clin Psychol.

[23] Cox JL, Holden JM, Sagovsky R (1987). Detection of postnatal depression. Development of the 10-item Edinburgh postnatal depression scale. Br J Psychiatry.

[24] Radoš SN, Tadinac M, Herman R (2018). Anxiety during pregnancy and postpartum: course, predictors and comorbidity with postpartum depression. Acta Clin Croat.

[B25] Silva RS, Azevedo R, Sampaio VS, Rodrigues KO, Fronza M (2021). Postpartum depression: a case-control study. J Matern Fetal Neonatal Med.

[26] Nijsten K, van der Minnen LM, Dean C, Bais JMJ, Ris-Stalpers C, van Eekelen R (2022). Depression, anxiety, and post-traumatic stress disorder symptoms after hyperemesis gravidarum: a prospective cohort study. J Matern Fetal Neonatal Med.

[27] Galletta MAK, Oliveira AMSS, Albertini JGL, Benute GG, Peres SV, Brizot ML (2022). Postpartum depressive symptoms of Brazilian women during the COVID-19 pandemic measured by the Edinburgh postnatal depression scale. J Affect Disord.

[28] Hollos P, Marchisella F, Coffey ET (2018). JNK Regulation of depression and anxiety. Brain Plast.

[29] Sekine Y, Tsuji S, Ikeda O, Sato N, Aoki N, Aoyama K (2006). Regulation of STAT3-mediated signaling by LMW-DSP2. Oncogene.

[B30] Zhao YT, Deng J, Liu HM, Wei JY, Fan HT, Liu M (2022). Adaptation of prelimbic cortex mediated by IL-6/STAT3/Acp5 pathway contributes to the comorbidity of neuropathic pain and depression in rats. J Neuroinflammation.

[31] Choi IY, Cho ML, Cho KO (2022). Interleukin-17A mediates hippocampal damage and aberrant neurogenesis contributing to epilepsy-associated anxiety. Front Mol Neurosci.

[32] Chuang HC, Chen YM, Hung WT, Li JP, Chen DY, Lan JL (2016). Downregulation of the phosphatase JKAP/DUSP22 in T cells as a potential new biomarker of systemic lupus erythematosus nephritis. Oncotarget.

[33] Gao W, Gao L, Yang F, Li Z (2022). Circulating JNK pathway-associated phosphatase: A novel biomarker correlates with Th17 cells, acute exacerbation risk, and severity in chronic obstructive pulmonary disease patients. J Clin Lab Anal.

[34] Wang C, Bai C, Mao C, Leng X, Wang F, Guo X (2022). JNK pathway-associated phosphatase illustrates low expression and negative correlations with inflammation, disease activity, and T-helper 17 cells in inflammatory bowel disease children. J Clin Lab Anal.

[35] Hwang S, Song KD, Lesourne R, Lee J, Pinkhasov J, Li L (2012). Reduced TCR signaling potential impairs negative selection but does not result in autoimmune disease. J Exp Med.

[36] Corsi-Zuelli F, Deakin B, de Lima MHF, Qureshi O, Barnes NM, Upthegrove R (2021). T regulatory cells as a potential therapeutic target in psychosis? Current challenges and future perspectives. Brain Behav Immun Health.

[37] Corsi-Zuelli F, Deakin B (2021). Impaired regulatory T cell control of astroglial overdrive and microglial pruning in schizophrenia. Neurosci Biobehav Rev.

[38] Matsumura K, Hamazaki K, Tsuchida A, Kasamatsu H, Inadera H, Japan E (2019). Education level and risk of postpartum depression: results from the Japan Environment and Children's Study (JECS). BMC Psychiatry.

[39] Chen Q, Zhuang J, Zuo R, Zheng H, Dang J, Wang Z (2022). Exploring associations between postpartum depression and oxytocin levels in cerebrospinal fluid, plasma and saliva. J Affect Disord.

